# Rate splitting with semantics as a generalized multi-access framework for intelligent reflecting surfaces

**DOI:** 10.1038/s41598-024-58422-y

**Published:** 2024-04-26

**Authors:** Senthil Kumar Jagatheesaperumal, Zhaohui Yang, Md. Rafiul Hassan, Mohammad Mehedi Hassan, Giancarlo Fortino

**Affiliations:** 1 Department of Electronics and Communication Engineering, Mepco Schlenk Engineering College, Sivakasi, 626005 India; 2 College of Information Science and Electronic Engineering, Zhejiang University, Hangzhou, 310027 China; 3 Zhejiang Provincial Key Lab of Information Processing, Communication and Networking (IPCAN), Zhejiang University, Hangzhou, 310007 China; 4 Department of Computer Science, Central Connecticut State University, New Britain, CT USA; 5 Department of Information Systems, College of Computer and Information Sciences, King Saud University, 11543 Riyadh, Saudi Arabia; 6 Department of Informatics, Modeling, Electronics, and Systems, University of Calabria, 87036 Rende, CS Italy

**Keywords:** Rate splitting, Multi-access, Semantics, Intelligent reflecting surfaces, Resource allocation, Information technology, Computer science

## Abstract

The rapid advancement of modern communication technologies necessitates the development of generalized multi-access frameworks and the continuous implementation of rate splitting, augmented with semantic awareness. This trend, coupled with the mounting pressure on wireless services, underscores the need for intelligent approaches to radio signal propagation. In response to these challenges, intelligent reflecting surfaces (IRS) have garnered significant attention for their ability to control data transmission systems in a goal-oriented and dynamic manner. This innovation is largely attributed to equitable resource allocation and the dynamic enhancement of network performance. However, the integration of rate-splitting multi-access (RSMA) architecture with semantic considerations imposes stringent requirements on IRS platforms to ensure seamless connectivity and broad coverage for a diverse user base without interference. Semantic communications hinge on a knowledge base-a centralized repository of integrated information related to the transmitted data-which becomes critically important in multi-antenna scenarios. This article proposes a novel set of design strategies for RSMA-IRS systems, enabled by reconfigurable intelligent surface synergizing with semantic communication principles. An experimental analysis is presented, demonstrating the effectiveness of these design guidelines in the context of Beyond 5G/6G communication systems. The RSMA-IRS model, infused with semantic communication, offers a promising solution for future wireless networks. Performance evaluations of the proposed approach reveal that, despite an increase in the number of users, the delay in the RSMA-IRS framework incorporating semantics is 2.94% less than that of a RSMA-IRS system without semantic integration.

## Introduction

As multi-antenna broadcasting becomes increasingly integral to modern communication systems, base stations equipped with multiple transmit antennas are capable of serving a broad range of users. The conventional multi-user linear precoding (MU-LP) architectures employed in 4G and 5G services rely on scheduling users with their encoded messages as streams, transmitting them over individual antennas. In such scenarios, each user is typically affected by residual interference, leading to imperfect channel state information at the transmitter (CSIT). Despite robust optimization efforts, novel approaches are essential to optimize the degrees of freedom (DoF) when utilizing multi-antennas. The prevalent misconceptions in applying rate splitting multiple access for 6G scenarios were discussed in^1^, where the role of rate splitting in next-generation networks was emphasized, along with the standards for establishing robust rate splitting-driven communication in 6G networks.

Rate splitting encompasses a superset of multiple access techniques, including Orthogonal Multiple Access (OMA), Non-Orthogonal Multiple Access (NOMA), Space Division Multiple Access (SDMA), and multicasting, thereby offering a universal means of access. Its versatility is also well-suited to handle a variety of network loads, whether overloaded or underloaded. Such flexibility provides a potent strategy for managing multi-user interference in the network. Optimizing the degree of freedom (DoF) is a key component in ensuring robustness in multi-access scenarios, capable of handling imperfect CSIT by addressing different network impairments. A novel rate-splitting strategy for uplink cognitive radio NOMA channels, aimed at maximizing user achievable rates, was developed by Liu et al.^2^. This approach allows secondary users to benefit without compromising the performance of primary users. The numerical results from this work indicate that the secondary user outage performance surpasses that of existing cooperative schemes.

In their study, Flores et al.^3^ proposed a robust power allocation methodology by incorporating insights from channel error variances to address imperfect CSIT, allowing for adaptive power adjustments. Their numerical results demonstrated that the sum rate of the rate splitting system, equipped with adaptive power allocation schemes, outperforms conventional multi-user systems, even with imperfect CSIT. Furthermore, Mishra et al.^4^ integrated rate splitting with time division duplex for downlink to assess spectral efficiency performance. Their transmission framework for multiple access via rate splitting includes power allocation schemes and precoder design to maximize network utility.

Moreover, rate splitting ensures lower complexity compared to other multi-access schemes. Beyond simplifying the scheduler and receiver complexities, it also reduces CSI feedback overhead. Rate splitting, augmented by cooperative relaying techniques, is employed to enhance coverage and transmission rates for users at the cell edge. This approach enables the decoding and distribution of the stream among intended users, thereby enhancing data rates, extending coverage, and improving channel strength. Importantly, with a focus on supporting Ultra-Reliable Low-Latency Communication (URLLC) services in 6G, rate splitting contributes to achieving low-latency transmission and enhanced throughput compared to other multi-access techniques. Additionally, rate splitting is instrumental in enhancing information security, such as in detecting the location of eavesdroppers. An instance of this application, involving Unmanned Aerial Vehicles (UAVs) for deploying energy-efficient and secure communication with maximized secrecy, was reported by Bastami et al.^5^. In various scenarios, this approach optimizes the imperfect information about channel states at the transmitter to address network allocation issues. Furthermore, by employing a two-tier intra-cell optimization strategy, optimal solutions are assured for multi-carrier UAV networks^6,7^.

Intelligent Reflecting Surface (IRS) technology enables smart and reconfigurable radio signal propagation, particularly for Beyond 5G/6G networks. An IRS can induce controlled phase changes and amplitude modifications to the signal incident on its large array of passive reflective elements. By deploying a dense array of IRS units, wireless networks can achieve flexible reconfiguration and desired signal distribution or realization. Additionally, the IRS offers a stringent mechanism to mitigate impairments in wireless channels, such as fading and interference, thereby significantly enhancing the reliability and capacity of future wireless communications^8,9^.

In^10^, the benefits of semantic communication within the Metaverse context were demonstrated, highlighting its potential for future industrial applications, particularly in enhancing digital twin deployment. The synergistic prospects of rate splitting and semantic communication could further elevate the capabilities of IRS in future wireless services. This paper distinguishes itself as the first to emphasize the impact of semantic communication on IRS in conjunction with rate splitting. In addition to reviewing state-of-the-art results on rate splitting in multi-access platforms, this paper provides a technical discussion on facilitating IRS deployment.

Focusing on the intricacies of RSMA-IRS integration, deployment challenges, and comparative analysis, this research poses several questions:

- How does RSMA-IRS integration enhance spectral efficiency, reduce interference, and improve wireless connectivity?
- What practical challenges and solutions exist for RSMA-IRS deployment, offering a holistic view of applicability?
- How does RSMA-IRS compare to other multi-access schemes in terms of connectivity, interference, and overall performance?
- What are the unique advantages, limitations, and trade-offs of RSMA-IRS in diverse network scenarios?

Based on these questions, our contributions to this work are summarized as follows:

- We develop a framework that integrates rate splitting with semantic awareness, formulating a resource allocation process that considers multiple access and semantic information.
- We incorporate semantics into this resource allocation process, augmenting it with a rate-splitting approach. This framework accounts for the location-dependent nature of Augmented Reality (AR) services, thereby allocating resources more efficiently.
- We validate the proposed framework through extensive simulations, demonstrating its superiority over benchmark schemes in key performance metrics such as transmitted power, delay, and sum-rate estimations, which are crucial for evaluating RSMA-IRS systems.

Deploying RSMA-IRS systems in real-world applications presents several challenges, including hardware requirements, scalability issues, and performance implications due to high user density and complex network environments. Innovative hardware designs, dynamic resource allocation techniques for scalability, and the incorporation of machine learning algorithms are imperative for adaptive RSMA-IRS systems. Additionally, the extension to real-world deployment issues underscores the importance of a dynamic and goal-oriented deployment strategy.

## Related Works

Numerous studies have investigated the deployment of RSMA in conjunction with supporting technologies for modern wireless communication standards. This section highlights several significant recent publications in this field.

Mao et al.^11^ provided a comprehensive summary of state-of-the-art research on rate splitting multiple access, detailing its taxonomy, architecture, and applications. They compared the performance and complexities associated with existing multi-access schemes and discussed research challenges, guiding future investigations into RSMA for Beyond 5G (B5G) systems. In a related study, Salem et al.^12^ developed precoding techniques for private messages in RSMA systems to enable secure communication features. Using Monte Carlo simulations, they demonstrated power allocations that balance trade-offs between secrecy and rate benefits in the system. Additionally, they analyzed the ergodic rates with novel power allocation strategies aimed at maximizing the system’s sum rate.

Lin et al.^13^ devised an optimization solution to maximize the sum rate in an RSMA system, catering to a wide range of IoT devices within the network. Addressing constraints related to antenna power and signal interference noise, they reduced the optimization problem to a rank-one problem, which was solved using an iterative penalty function algorithm. Their findings indicated that this optimization solution effectively suppresses interferences and enhances the system’s sum rate. Another innovative approach, proposed by Xu et al.^14^, integrates RSMA with dual-functional radar communication for multi-antenna joint radar systems in B5G applications. This methodology employs a unified interference management strategy, proving efficient in both user communication and radar signal detection. The system simultaneously considers factors such as sum rate, mean square error, and power constraints. Table 1 presents a comparative analysis of various multi-access schemes, elucidating the strengths and limitations of the RSMA-RIS framework in comparison to other methodologies.

**Table 1:** Comparative Analysis of Multi-Access Schemes, with RSMA-RIS Framework.

Multi-access scheme	Key characteristics	Advantages	Limitations
Time division multiple access (TDMA)	Shares a single carrier frequency with multiple users.	Simple implementation, low latency	Inefficient spectrum utilization, scalability issues
Frequency division multiple access (FDMA)	Channel capacity is shared among users to communicate independently	Reduce interference, predictable performance	Limited flexibility, susceptibility to frequency collisions
Cognitive radio scheme	Dynamic spectrum access, learning algorithms	Adaptive to changing environments, efficient spectrum use	Complexity in cognitive decision-making, Spectrum sensing challenges
Spatial modulation scheme	Spatial signal encoding, multiple antennas	Efficient use of spatial dimensions, Increased data rates	Susceptible to channel fading, limited coverage range
Non-orthogonal multiple access (NOMA)	Enhanced secrecy capacity, interference cancellation	Increased spectral efficiency, support for diverse users	Complexity in power allocation, interference management challenges
RSMA-RIS framework	Rate-splitting, Semantic-aware networking, IRS usage	Enhanced spectral efficiency, reduced interference	Complexity in IRS deployment, hardware demands

In another study focusing on a specific RSMA scenario in cloud radio access networks, Ahmad et al.^15^ address the optimization of stochastic coordinated beamforming to maximize the ergodic sum rate within the network. Their approach involves robust and scalable rate splitting, where the statistical channel state information (CSI) of users influences the decoding characteristics. This influence is linearly proportional to the number of users in the network. Through this method, stochastic non-convex optimal solutions are achieved, demonstrating improved efficiency compared to Non-Orthogonal Multiple Access (NOMA) schemes. Additionally, Flores et al.^16^ employed Tomlinson-Harashima precoders, considering multi-antenna receivers, to analyze rate-splitting with data streams. Their developed method enhances the performance of the sum rate by incorporating a multi-branch structure in the rate-splitting architecture. The analyses conducted focused on assessing the signal-to-noise ratio and interference at the receiver. The results indicated that the performance of their approach is superior compared to conventional linear approaches.

Orthogonal and non-orthogonal rate-splitting, as discussed by Gamal et al.^17^, have been identified as key multiple access strategies for cognitive radio networks, primarily focusing on enhancing spectral efficiency. The study addresses implementation challenges related to enhancing spectral resources through enabling techniques, demonstrating that RSMA provides better multiplexing gain and addresses inefficiencies in spectral resource utilization.

Salem et al.^18^ argue that rate-splitting is a promising solution for exploiting and suppressing interference, especially when considering finite constellations. They employed zero-forcing and closed-form precoding strategies to develop an analytical framework for the ergodic sum rate. Additionally, the study examines power allocation strategies and compares them with conventional precoding dependent on rate splitting.

The work by Bansal et al.^19^ investigates a specific on-off scheme for controlling IRSs with practical phase shifts. By deriving closed-form expressions for near-proximity and cell-edge users, the study analyzes user performance, noting that the introduction of rate-splitting-based IRS enhances cell user performance and addresses the impacts of imperfect channel states. Increasing the number of reflecting elements in the network can significantly reduce estimation errors and improve network performance.

De et al.^20^ introduces the interplay of RSMA with IRS, highlighting three crucial enhancements for Beyond 5G (B5G) use cases. This research offers insights with numerical evaluations, facilitating flexible interference management and potentially higher energy and spectral efficiency.

Zhao et al.^21^ focus on enhancing user relaying through cooperative rate splitting driven by RIS. They characterize the transmission framework of cooperative rate splitting by addressing resource allocation. The study optimizes common rate allocation and transmits beamforming along with RIS phases iteratively, aiming to maximize the minimum rate among users. Weinberger et al.^22^ address interference mitigation through the rate-splitting strategy in IRS environments. Their work ensures Quality of Service (QoS) constraints for users, focusing on minimized transmission power usage, power savings, and yield improvements, particularly in cloud Radio Access Network (RAN) environments to optimize the positive influence on mitigating interference in transmissions, often affected by reflections.

In^23^, the authors demonstrate a rate-splitting-based IRS-driven cognitive radio system that ensures the QoS of primary users by setting an intolerable threshold for interference. This approach also enhances the outage performance of secondary users with optimized power allocation for transmission and better successive interference cancellation decoding orders. Huang et al.^24^ focus on a multi-user platform via IRS, utilizing a Virtual Reality (VR) streaming strategy for high-resolution 360-degree video streams. The IRS primarily aids in mitigating performance bottlenecks for all users engaged in rate splitting while decoding transmitted messages. Furthermore, they deployed a deep learning-based policy gradient strategy, using imitation learning and Deep Reinforcement Learning (DRL), to optimize IRS phase shifts, thereby addressing video tiles through bitrate selection.

**Figure 1:**
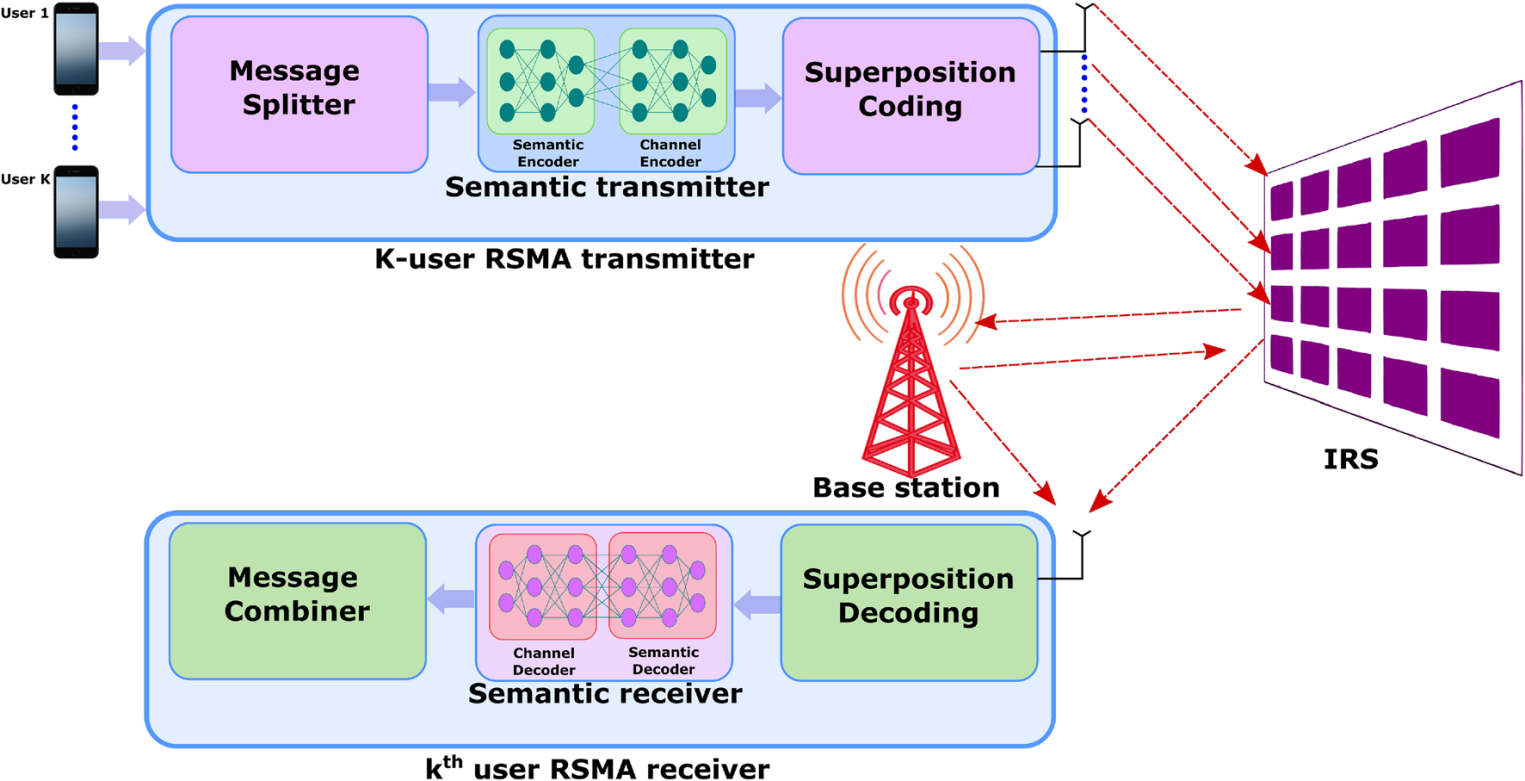
A K-user RSMA transmitter with semantic communication in an IRS-enabled scenario (The red arrows represent the incident and reflection signals from the IRS plane).

Energy-efficient implementations of RSMA in conjunction with RIS have been explored, particularly focusing on the control of RIS phase shifts to adjust the beamforming characteristics of base stations, as discussed by Yang et al.^25^. It is noteworthy that both RSMA and RIS aim to develop optimized solutions for future wireless communication services, offering automated and flexible connectivity, as well as QoS and Quality of Experience (QoE) provisioning for end-users. A RIS-based RSMA approach for 6G multi-access networks can significantly enhance performance metrics, as shown in Li et al.^26^. However, addressing challenges in next-generation multiple access strategies necessitates considering imperfect channel conditions. Recent advancements in semantics-oriented communication are expected to mitigate channel imperfections by enhancing transmission and reception quality through robust knowledge base encoding mechanisms. The practical challenge of effective semantic representation can be tackled by utilizing a semantic knowledge base to guide communication. In such a base, source messages are characterized in low-dimensional subspaces, maintaining their intended meaning and significantly improving communication efficiency.

The concept of a fully connected RIS-based RSMA was introduced by Fang et al.^27^ for multi-user transmission via multiple antennas. This approach significantly improves spectral efficiency compared to schemes without RIS, setting a new benchmark for linearly precoded wireless networks. Gao et al.^28^ designed a joint optimization solution to maximize the minimum secrecy rate, thereby securing the RSMA-IRS system against eavesdroppers. Their findings highlight the enhanced max-min secrecy rate in RSMA systems equipped with IRS elements compared to those without.

Liu et al.^29^ demonstrated the use of IRS in aiding spectrum users in cognitive rate-split multiple access systems. Here, secondary users engage in rate splitting, transmitting signals to the base station through both IRS reflection channels and direct links. Optimized transmission rate allocation strategies were employed to achieve the maximum range of allowable achievable rates. Such IRS-driven cognitive RSMA results in improved outage probability for secondary users in the network.

For realizing sum-rate maximization in multiple RIS-driven scenarios, dynamic user clustering and RSMA strategies are employed, as detailed by Katwe et al.^30^. This approach involves considering uplink users and beamforming design in a joint optimization strategy for power allocation within effective low-cost frameworks. Additionally, a unified solution was developed to assess user fairness and the correlation among RIS elements, aiming to enhance the capabilities of millimeter-wave systems.

The study by Hassan et al.^31^ focuses on optimizing target radiation patterns in a 2D plane of stacked intelligent metasurfaces, demonstrating efficient power concentration using three layers for continuous coefficients and nine layers for discrete coefficients. This approach achieves a nearly 50% increase in power ratio compared to a single-layer stacked intelligent metasurface. The concept of perpetual RISs, as explored in Ntontin et al.^32^, offers a highly energy-efficient solution for 6G networks through wireless energy harvesting to sustain their operation. The authors outlined the RIS controller architecture and energy-harvesting protocols, exploring their performance in communication scenarios and addressing research challenges for large-scale perpetual RIS networks.

## Semantic framework for Intelligent Reflecting Surfaces

This section focuses on formulating a problem essential for developing a semantic framework enabling IRSs to comprehend their environment and respond effectively. The challenge lies in creating a robust and reliable semantic framework that can manage the complex and dynamic nature of the environments where IRSs operate.

Consider a wireless communication system comprising multiple users and an IRS that reflects signals from the base station (BS) to these users. The IRS can modify the phase shifts of the reflected signal to enhance signal quality and maximize system throughput. The objective is to devise a resource allocation algorithm that considers the semantic information of the users and employs rate splitting to enhance system performance.

Figure 1 illustrates a scenario involving a group of *K* users with B5G/6G-enabled customer premises equipment participating in a secure and robust information exchange mission. In this scenario, *K* users transmit data to a base station, each with a specific data quota, sharing the same wireless resources. The RSMA transmitter enables users to divide their transmission rate into two streams: a common stream shared among all users and a private stream exclusive to each user. The transmission incorporates an IRS system, comprising reflective surfaces that manipulate wireless signals to improve signal quality and, consequently, wireless communication performance. This communication process accounts for the location-dependent nature of wireless communication, facilitated by semantic information, optimizing the allocation of wireless resources among users based on their physical locations and wireless channel characteristics.

Mao et al.^33^ recently explored the application of RIS in terahertz (THz) communications in a multi-user system. Their approach utilizes index modulation on an array-of-subarrays structure, thereby enhancing spectrum and energy efficiencies. RIS-assisted transmission is noted for its high spectral and energy efficiency, particularly when combined with spatial modulation techniques^34^. Such systems are increasingly prevalent in Wireless Sensor Networks (WSNs), cognitive radio applications, and relay networks. Notably, there is significant potential in integrating the RSMA transceiver with a semantic communication module. Specifically, the signal reflected to the *k*th user from the IRS environment is incorporated into the RSMA transceiver’s semantic decoding unit. The signal from the base station on a particular channel is denoted as $\textbf{x}\in \mathbb {C}^{M\times 1}$. The signal received by the *k*th user near the IRS is represented by Eq. (1), as follows:

1 $$\begin{aligned} y_{k} = \textbf{h}^{H}_{k}\textbf{x} + w_{k}, \end{aligned}$$

where *M* is the number of base station antennas, *N* is the number of reflecting surfaces, and $\textbf{h}_{k}\in \mathbb {C}^{M\times 1}$ represents the channel between *k*th receiver and the base station and $w_{k}\in \mathcal{C}\mathcal{N}(0,1)$ is the AWGN at the *k*th user. In the aforementioned scenario, the signal received at user-$k^{'}$ is shown in Eq. (2):

2 $$\begin{aligned} y_{k^{'}} = \textbf{h}^{H}_{k^{'}}\textbf{Z}_{k}\textbf{G}_{k}\textbf{x} + w_{k^{'}}. \end{aligned}$$

where $\textbf{G}_{k}\in \mathbb {C}^{N\times M}$ and $\textbf{h}_{k^{'}}\in \mathbb {C}^{N\times 1}$are the channels between the base station to *k*th IRS and *k*th IRS to $k^{'}$th user respectively. To establish finite beamforming for IRS, the effective channel vector of the users through an on-off keying strategy. The vector for multiple users is represented as a matrix, where the diagonal element is either 1 (on) or 0 (off) and is represented as $\varvec{\Theta }$. With certain constraints on the choice of vector elements, the $\varvec{\Theta }$ is defined as $\textbf{Z} = \text {diag}\{\textbf{a}_{p}\}$, where $\textbf{a}_{p}$ is the *p*th column of the matrix $\textbf{A}$.

Semantic-oriented communication can significantly enhance efficiency and transmission quality by leveraging various source modalities from the multi-access environment. The knowledge base in semantic communication, encompassing formats such as knowledge graphs, trained models, and databases, plays a crucial role. The semantic transmitter processes the source data, extracting semantic features through the knowledge base. This is followed by the semantic channel, which adeptly adapts the signal for effective transmission. The semantic receiver then employs semantic fusion techniques to intelligently reconstruct the data. In the context of the RSMA-RIS transceiver, the semantic metrics ensure the provision of task-specific similarity. This approach facilitates the transmission of only the essential components of the user’s message, based on the core knowledge base. The semantic segmentation, compression, and encoding modules are heavily dependent on the specific task at hand. Algorithm 1 delineates the resource allocation process for users, taking into account the base station antennas and the role of IRS in establishing semantic communication.

**Algorithm 1:**
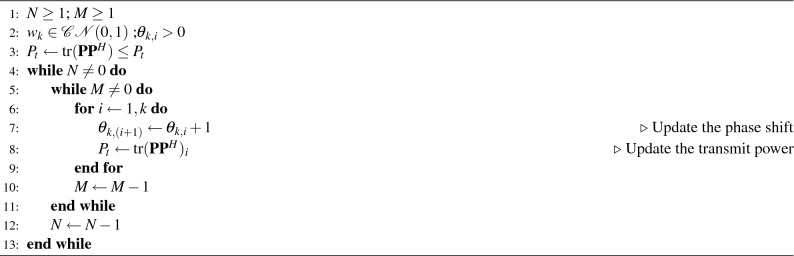
Resource Allocation for Multi-Access IRS.

## Performance evaluation

In this section, we describe the simulation settings and analyze the performance of the RSMA-based IRS system, augmented with semantic-aided communication.

The MATLAB simulations were conducted on a computing terminal featuring an Intel Core i9 processor, 32 GB of RAM, and an NVIDIA RTX 3080 GPU. A realistic channel model was utilized to emulate wireless communication scenarios involving the transmitter, IRS, and receiver. These scenarios varied in terms of the level of semantic information and the dynamic distances between the IRS and the communicating parties. Key performance metrics, including transmitted power, delay, and sum-rate estimations, were employed to assess the effectiveness of the RSMA-based IRS system enhanced with semantic-aided communication.

**Figure 2:**
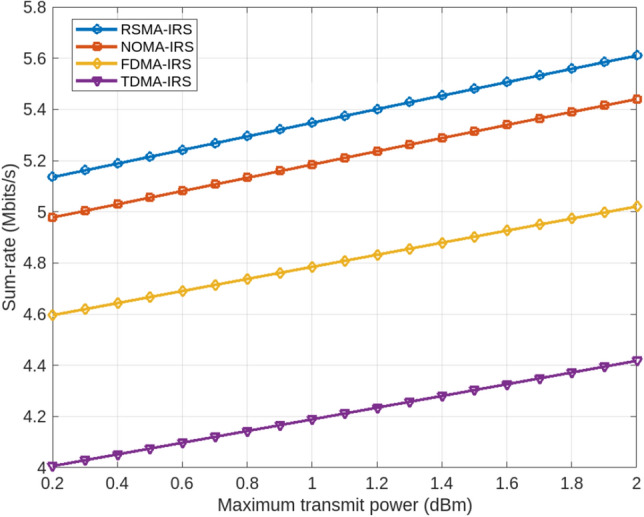
Comparative analysis of Maximum Transmit power over Sum-rate for RSMA-IRS and other Multi-access Schemes.

In our evaluation of the RSMA performance with semantic enhancements, we analyzed the network’s performance in comparison to other multi-access schemes such as NOMA (Non-Orthogonal Multiple Access), FDMA (Frequency Division Multiple Access), and TDMA (Time Division Multiple Access). Regardless of the perfection in Channel State Information at the Transmitter (CSIT), the rate-splitting scheme demonstrates superior spectral efficiency and optimized sum-Degrees of Freedom (sum-DOF) compared to these schemes. Additionally, its trade-off can be fine-tuned to surpass other multi-access methods in terms of energy efficiency, regardless of network loads and user engagement. In multi-user scenarios, there is a demand for better QoS with fairness in resource allocation decisions. Rate splitting, in this context, enhances performance gains and effectively meets user rate requirements under QoS constraints. Even in the presence of weaker channel strengths, rate splitting ensures improved QoS and fairer policies in managing user requirements. Figure 2 presents a comparative analysis of maximum transmit power versus sum-rate for RSMA and other multi-access schemes, highlighting the beneficial impact of incorporating RIS with semantic awareness in the network, thereby offering superior characteristics compared to alternative schemes.

The performance evaluation of maximum transmit power relative to sum-rate for RSMA is instrumental in identifying the strengths and limitations of the proposed scheme under various network scenarios. This analysis also elucidates how the scheme compares and interacts with other multi-access systems and the potential for optimizing system energy efficiency, spectral efficiency, and sum-DOF. Moreover, it assesses how effectively rate splitting contributes to performance gains while ensuring fairness in resource allocation and addressing user rate requirements under QoS constraints. The inclusion of RIS with semantic awareness in the network is shown to offer superior characteristics over other multi-access schemes, ensuring better QoS and fairer policies, even in scenarios characterized by weaker channel strengths.

Beyond the standard evaluation parameters, additional considerations included the following: Spectral efficiency, measured in bits per second per Hertz (bps/Hz), highlights the effective utilization of the available spectrum for data transmission. The interference reduction ratio quantifies the RSMA-based IRS’s ability, augmented with semantic-aided communication, to mitigate interference and enhance overall signal quality. Latency reduction, measured in milliseconds (ms), focuses on the communication latency achieved by the RSMA-RIS platform, contributing to faster data transfer. Power consumption, measured in milliwatts (mW), evaluates the power efficiency of the RSMA-RIS system, a critical factor for sustainable and energy-efficient wireless communication.

**Figure 3:**
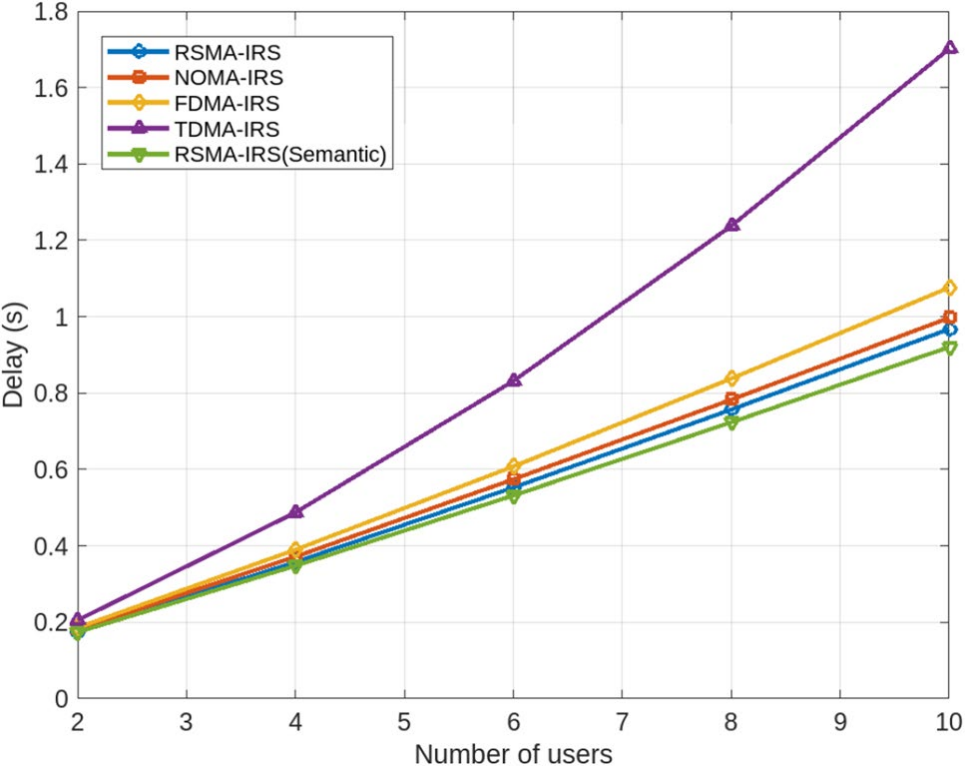
Delay characteristics concerning Number of users for RSMA-IRS with and without semantics and other Multi-access Schemes.

Modern communication paradigms are increasingly focusing on goal-oriented and semantic aspects, which are instrumental in identifying the most crucial information for the transceiver. In this context, the communication process within the channel is closely aligned with the semantic information present at the transmission end. The semantic features in the source account for imperfections in the wireless channel, enabling the extraction, compression, and synthesis of data, regardless of fading, noise, and interference. Furthermore, semantic communication incorporates intelligent means of information exchange, including encryption-based security measures to prevent unauthorized decoding by eavesdroppers. Additionally, semantic receivers in intelligent radios are adept at estimating channel state information and discerning multi-user signals, effectively separating individual user signals within the network.

**Figure 4:**
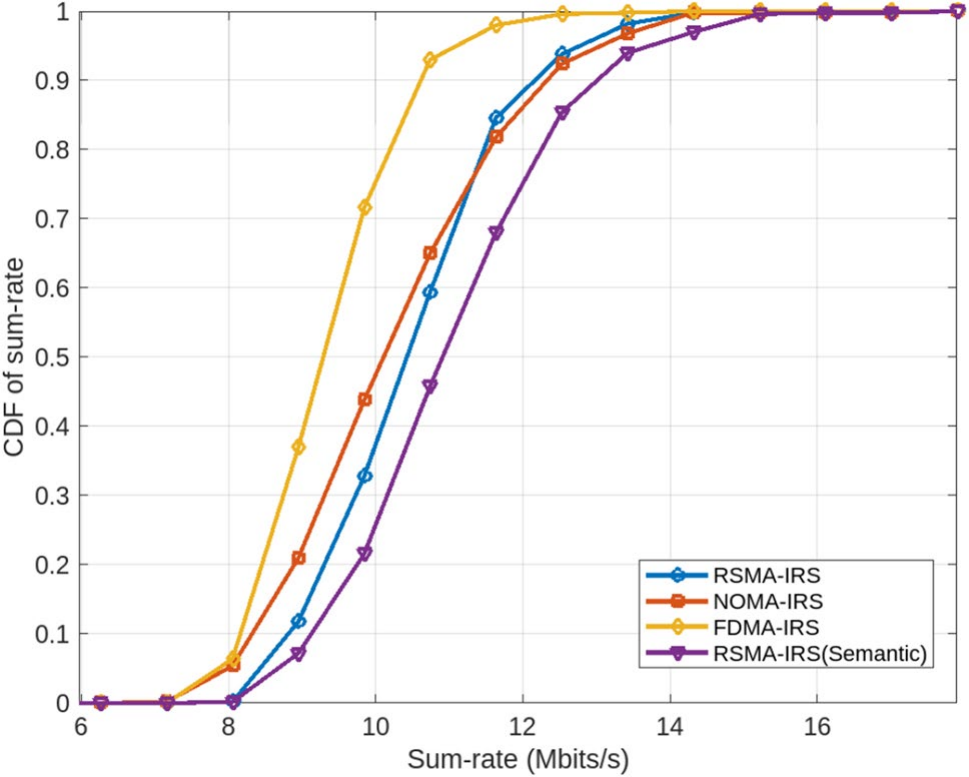
CDF of sum-rate estimation in association with sum-rate for RSMA-IRS with and without semantics along with other Multi-access Schemes.

Evaluating the delay characteristics of RSMA-IRS is pivotal in ensuring the suitability of the proposed scheme for modern applications, where low latency and high reliability are paramount. This is especially critical in contexts such as augmented reality, remote surgery, and autonomous vehicles. Since the number of users in a network can significantly impact the delay in data transmission and reception, leading to potential performance degradation, it is essential to assess the delay characteristics of RSMA-IRS in various scenarios. These evaluations are vital for comparing RSMA-IRS with other multi-access schemes. Additionally, analyzing delay can offer insights into the interaction of the proposed scheme with other key parameters like packet loss, throughput, and jitter, which are influential in determining the system’s overall performance. To illustrate this, Fig. 3 compares the delay characteristics of RSMA-RIS with other multi-access schemes, considering the involvement of different numbers of users.

IRS technology, known for its economic, efficient, and effective spectral usage, is increasingly being adopted in multi-access networks. The precise beams reflected by IRS surfaces allow base stations to connect multiple users with fewer active antennas, without compromising the QoS for network users. This capability of IRS helps in reducing the number of active antennas required, exploiting the sharp beams generated from the reflecting surfaces. To further understand the performance of the RSMA-RIS framework in association with integrated semantics, Fig. 4 presents the Cumulative Distribution Function (CDF) of the sum-rate estimation, comparing it with other multi-access techniques.

Despite the advantages offered by IRS in terms of cost, effectiveness, and spectrum consumption in multi-access networks, there are inherent limitations. One such limitation is the reliance on fine-grain beams from IRS surfaces, which necessitates fewer active antennas at base stations. However, this dependence on precise beams can pose challenges in scenarios with obstructions to direct line-of-sight or in dynamic environmental conditions. Additionally, the efficacy of IRS in reducing the total number of active antennas may vary based on the specifics of the communication environment.

## Conclusion

This paper examines the implementation of semantic communication in an RSMA transceiver within an IRS environment. The semantic features are employed to distill the core knowledge base content from users, enabling the RSMA transmitter to address signaling overhead while achieving better link budget gains. The multi-carrier RSMA approach is characterized by low latency and reduced power constraints, which are further refined through the IRS frameworks within the network. To enhance overall network performance and provide improved QoS to users, semantic-aware networking is utilized to balance the expected benefits. Additionally, the results were analyzed to understand the positive impact of the RSMA-RIS platform relative to other multi-access schemes in conjunction with the RIS environment. The observations indicate that RSMA offers a superior design strategy for future Beyond 5G/6G wireless communication services.

## Data Availability

All data generated or analysed during this study are included in this published article.
